# RNA-sequencing reveals genome-wide long non-coding RNAs profiling associated with early development of diabetic nephropathy

**DOI:** 10.18632/oncotarget.22405

**Published:** 2017-11-11

**Authors:** Wanxin Tang, Dongmei Zhang, Xiaoke Ma

**Affiliations:** ^1^ Department of Nephrology, West China Hospital, Sichuan University, Chengdu, Sichuan, China; ^2^ School of Computer Science and Technology, Xidian University, Xi'an, Shaanxi, China

**Keywords:** long non-coding RNA, kidney, nephropathy, gene expression, network biology

## Abstract

**Background:**

Diabetic nephropathy (DN) seriously threatens the lives of patients, and the mechanism of DN remains largely unknown because of the complex regulation between long non-coding RNA (lncRNA) and protein-coding genes. In early development of diabetic nephropathy (DN), pathogenesis remains largely unknown.

**Results:**

We used RNA-sequencing to profile protein-coding and lncRNA gene transcriptome of mouse kidney proximal tubular cells during early stage of DN at various time points. Over 7000 protein-coding and lncRNA genes were differentially expressed, and most of them were time-specific. Nearly 40% of lncRNA genes overlapped with functional element signals using CHIP-Seq data from ENCODE database. Disease progression was characterized by lncRNA expression patterns, rather than protein-coding genes, indicating that the lncRNA genes are potential biomarkers for DN. For gene ontologies related to kidney, enrichment was observed in protein-coding genes co-expressed with neighboring lncRNA genes. Based on protein-coding and lncRNA gene profiles, clustering analysis reveals dynamic expression patterns for kidney, suggesting that they are highly correlated during disease progression. To evaluate translation of mouse model to human conditions, we experimentally validated orthologous genes in human cells *in vitro* diabetic model. In mouse model, most gene expression patterns were repeated in human cell lines.

**Conclusions:**

These results define dynamic transcriptome and novel functional roles for lncRNAs in diabetic kidney cells; these roles may result in lncRNA-based diagnosis and therapies for DN.

## INTRODUCTION

Diabetic nephropathy (DN) is major serious complication of diabetes and is the most common cause of end-stage renal disease with poor prognosis and high cost for therapy [1]. However, considering incomplete understanding of DN pathogenesis, early efficacious diagnosis and treatment are still unresolved issues. Emerging evidence show that proximal tubular cells (PTCs) play critical role in onset and progression of DN. Dimensions and function of proximal tubule increase in response to higher glucose reabsorption, which is caused by increased glomerular filtration of glucose [2], inducing glomerular hyperfiltration through tubuloglomerular feedback [3]. In response to hyperglycemia, PTCs exhibit, early behaviors, such as cell cycle arrest, hypertrophy, and senescence phonotype [4], which are linked to late inflammation, fibrosis, and apoptosis [5]. Kidney injuries are worsened by underlying pathogenic mechanisms for PTC metabolic disorder and abnormal response involving numerous genes and their precise transcriptional regulation networks. However, available information is insufficient to describe changes in genes and regulation during progress of DN.

Long non-coding RNAs (lncRNAs) are novel class of functional RNAs; these transcripts measure more than 200 nt and do not code for proteins. lncRNAs and protein-coding transcripts exhibit many similarities, which are as follows: a) presence of 5′ cap and 3′ poly adenosine tail structures; b) transcribed by RNA-polymerase (Pol) II; c) can be spliced at canonical splicing sites [6]. Growing knowledge suggests that lncRNA may play critical role in growth, development, senescence, and disease [7, 8]. And many computational tools has been developed [9–11]. lncRNAs have the following primary functions on gene expression: as regulators of transcription via chromatin modulation [12] and epigenetic modification [13], as regulators of mRNA processing via influence splicing patterns of mRNAs [14], and as modulators of post-transcriptional control [15]. Complex functions of lncRNA are far beyond current understanding and require further characterization.

Strong correlation between LncRNA and diabetes was reported in recent literature. Morán et al. identified more than 1100 lncRNAs in human islets and several lncRNAs, which were dysregulated in islets from *type 2 diabetes* patients [16]. Data from study by Xu et al. showed that silencing lncRNA-nc021972 alleviated activation of P2×7 receptor and subsequent tumor necrosis factor-α and interleukin-6 release in *in vitro* DN models [17]. In recent studies, evidence also demonstrated involvement of lncRNAs in regulation of pathologic genes associated with DN. Surveys by Alvarez et al. showed that in mesangial cells, lncRNA plasmacytoma variant translocation 1 increases plasminogen activator inhibitor 1 and transforming growth factor beta 1, which are two primary contributors to extracellular matrix accumulation in glomeruli under hyperglycemic conditions [18]. Long et al. reported that peroxisome proliferator-activated receptor gamma coactivator alpha (PGC-1α) is functionally regulated by lncRNA taurine-upregulated gene 1 (Tug1). Direct interaction between PGC-1α and Tug1 can modulate mitochondrial bioenergetics in podocytes in DN models [19]. Wang et al. discovered that lncRNA CYP4B1-PS1-001 and ENSMUST00000147869 were significantly downregulated in response to early DN in db/db mice, whereas overexpression of two lncRNAs inhibited proliferation and fibrosis of mesangial cells [20, 21].

However, above sporadic studies provided insufficient information in genomic changes of lncRNA profiles for specific kidney cells during DN progression. Therefore, genome-wide discovery of lncRNA is needed to identify new concepts and opportunities for profoundly understanding pathogenesis and novel treatment of DN. Next-generation sequencing (NGS) technologies provide edge-cutting method for gene expression research, especially those including lncRNAs under pathophysiological conditions [22]. Thus, aim of this study was threefold. First, to discover genome-wide mRNA and lncRNA profiles of fresh isolated PTCs in animal model during dynamic progression of DN by using NGS. Second, to identify novel lncRNAs, evaluate importance of LncRNA and predict relationship between mRNA and lncRNA during dynamic disease progression by using algorithms. Third, to validate the identified novel RNAs may also play role in pathogenesis of human DN. Our first exploration of genome-wide lncRNAs in DN progression may drive discovery of new early biomarkers and novel therapeutic strategies.

## RESULTS

### Experiments workflow and animal models

**Figure 1:**
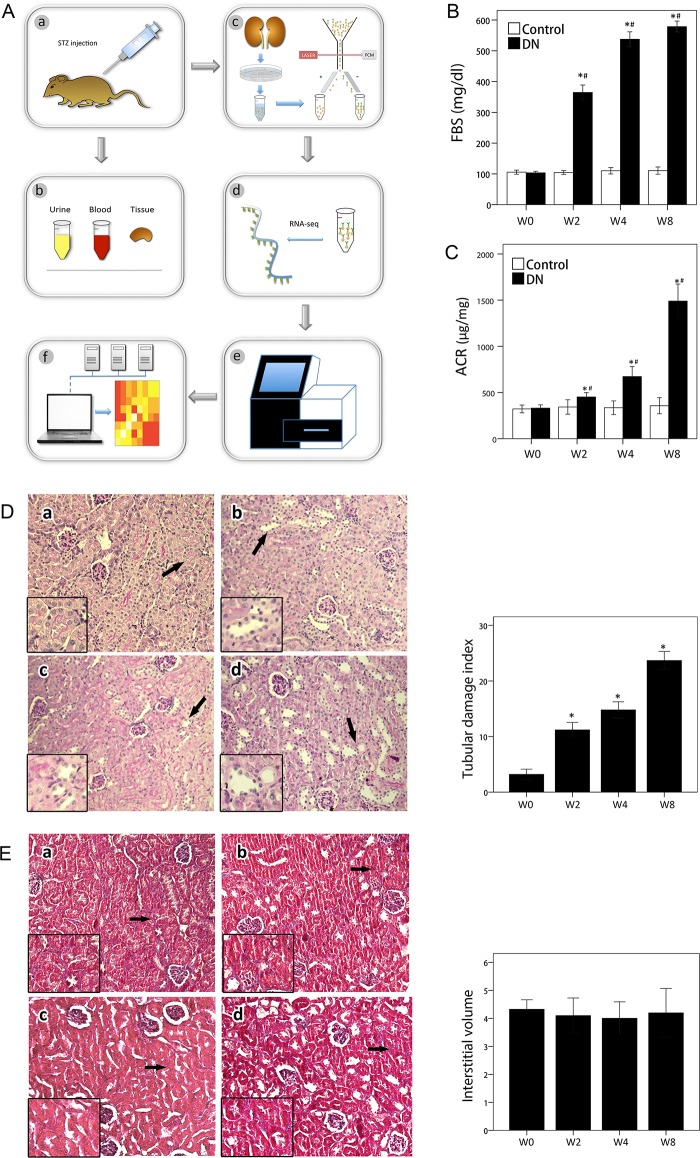
Workflow and phenotypes of animal models **(A)** Workflow of experiments. a. Construction of animal models; b. examination of phenotype; c. PTC isolation; d. libraries for preparation of RNA-sequencing; e. next-generation sequencing; f. bio-information analysis. **(B)** Fasting blood sugar over time. Data are represented as mean ±SEM. ^*^p < 0.05 by *t*-test versus control at 0 week. ^#^*p* < 0.05 by *t*-test versus control at the same week. *n* = 6 for each group. **(C)** ACR over time. Data are represented as mean ±SEM. ^*^p < 0.05 by *t*-test versus control at 0 week. ^#^*p* < 0.05 by *t*-test versus control at the same week. *n* = 6 for each group. **(D)** Left: PAS-stained sections in each group. Images display kidney sections of each group at 200× magnification. White arrow indicates normal tubule, and black arrow indicates injured tubule. Views with high magnification are shown at lower left. Time course are labeled as a, b, c, and d; letters correspond to 0, 2, 4, and 8 weeks. Right: Tubule injuries were evaluated for widened lumen, atrophy, or thickened basement membranes. Tubular injuries are indicated with arrows. Bar graph shows TDI for each group. Data are represented as mean ±SEM. ^*^p < 0.05 by *t*-test versus control in 0 week. *n* = 6 for each group. **(E)** Left: Masson-trichrome-stained kidney sections in each group. Images display kidney sections of each group at 200× magnification. White arrow indicates normal tubule, and black arrow indicates injured tubule. Views with high magnification are shown at lower left. Time courses are labeled as a, b, c, and d, corresponding to 0, 2, 4, and 8 weeks. Right: Bar graph shows relative interstitial volume for each group. Data are represented as mean ±SEM. *n* = 6 for each group.

The workflow was shown in Figure 1-A and the purity of sorted cells was shown in Supplementary Figure 1. All mice in model group developed remarkably high blood glucose (>300 mg/dl) two weeks after first STZ injection (Figure 1-B), indicating successful establishment of mouse diabetes models for subsequent experiments. Urine albumin–to–urine creatinine ratio (ACR) level was also gradient-increased two weeks after STZ injection and peaked at week 8 (Figure 1-C), indicating that kidney injury appeared in early stage of mouse diabetes models. In PAS staining at 2, 4, and 8 weeks of our study, tubular tissue exhibited enlarged lumen, bared cells, and reduced or lost microvilli at different degrees. Statistical difference of TDI with value obtained of 0 w has reached (Figure 1-D), indicating existence of tubular injury in DN development [23]. Tubulointerstitium fibrosis is late-stage character lesion of DN. Masson trichrome–staining showed absence of fibrosis of tubulointerstitium (Figure 1-E). Results indicated that DN was in early stage.

### Systematic profiling of mRNAs during early development of DN

We performed factorial RNA-Seq study to monitor transcriptome for mouse kidney PTCs during early development of DN. Specifically, we monitored disease progression at four time points after inducing DN: 0, 2, 4, and 8 weeks (denoted by W0/W2/W4/W8). For each time point, two biological replicate RNA-Seq data were generated.

Using unique mapped reads to estimate expression levels of mRNA genes, we identified 21,599 mRNA genes, of which 9,625 were expressed at FPKM value≥ 1 in at least one sample. Quantification results are consistent with strong correlation of gene expression between replicate samples (mean correlation coefficient = 0.98; mean standard deviation = 0.006; Supplementary Figure 2).

**Figure 2:**
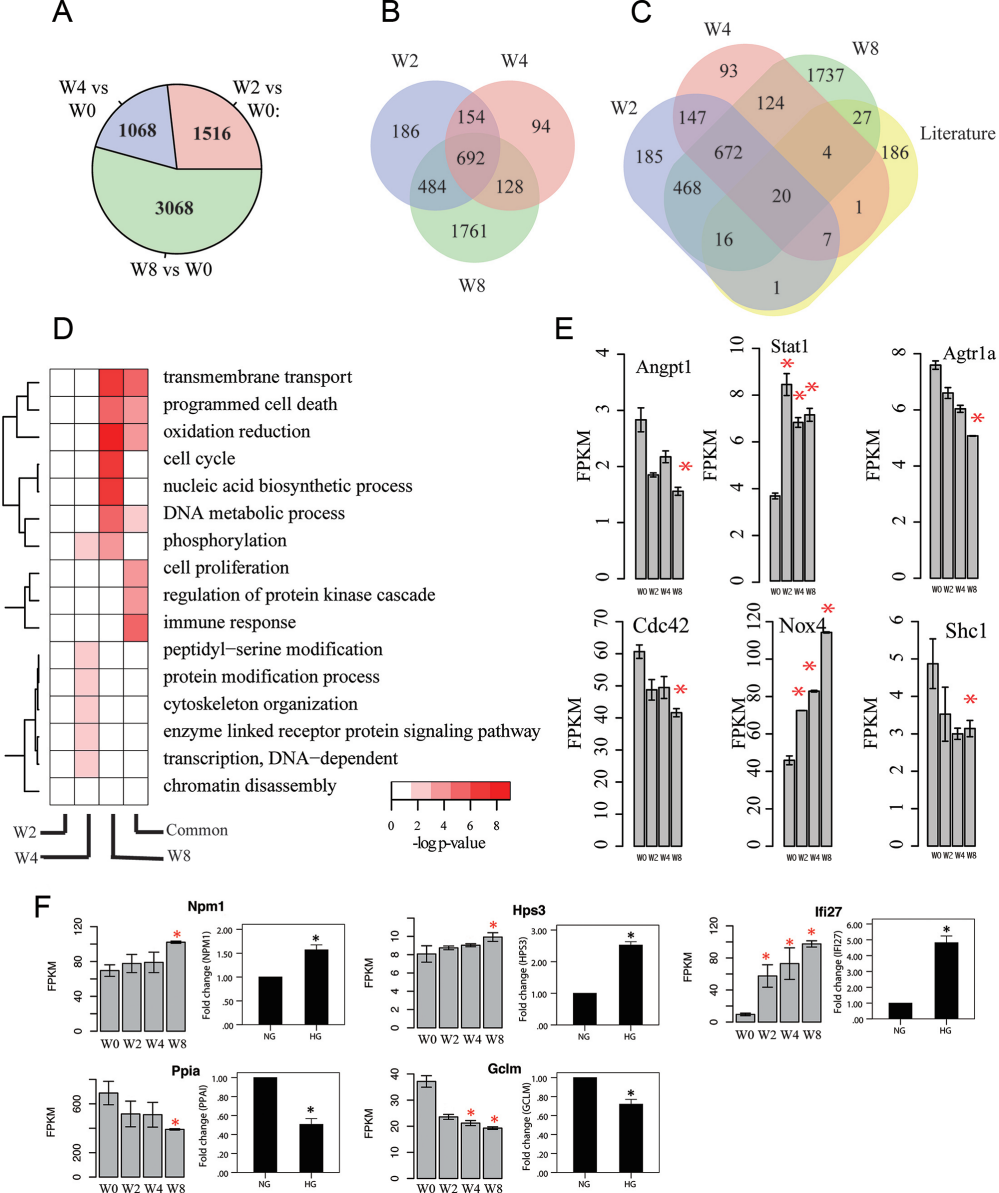
Transcriptome profiles of mRNAs associated with disease progression **(A)** Number of differentially expressed mRNA genes at various time points compared with control (W0). *P*-value cutoff is 0.05. **(B)** Venn diagram of differentially expressed mRNA genes at different times. **(C)** Overlapping between differentially expressed mRNA genes and reported mRNA genes associated with kidney disease progression. **(D)** Gene ontology enrichment for week-specific and common differentially expressed mRNA genes, presented as -log10 hypergeometric *P*-value for enrichment. **(E)** Gene expression of some key genes that are highly related to kidney. y-axis denotes FPKM value, x-axis is for four time points, and error bar is standard deviation, ^*^ indicates *p*-value < 0.05 by comparison with W0. **(F)** Validation for differentially expressed mRNAs in human PTCs (HK2)Left portion: Results from RNA-seq in mouse DN models (^*^p<0.05 versus W0). Right portion: Results from qPCR *in vitro* diabetic models. NG, cultured with normal glucose; HG, cultured with 30 mM glucose for 48 h.^*^p<0.05 versus NG, *n*=3.

Taking W0 as control, we identified differentially expressed protein-coding genes (Materials). We observed largest number of differentially expressed genes in W8 versus W0 comparison (*N*=3068, FDR <0.05) followed by W4 (W2) versus W0 comparison (*N*=1068 and 1516) (Figure 2-A), suggesting that more disturbed transcriptome of mRNAs is associated with progression of kidney disease. Then, we checked overlapping among differentially expressed genes at different time points (Figure 2-B). W2 and W4 have time-specific genes of 186 and 94, respectively. By contrast, W8 has 1761 specific differentially expressed genes. This result suggests very modest disturbance to transcriptome between W2 and W4.

To further characterize differentially expressed genes obtained from RNA-Seq analysis, we manually curated gene list associated with kidney by literature research, where 262 protein-coding genes were included (Supplementary Table 3). And, 76 of them are differentially expressed based on our analysis (Figure 2-C). For example, *Angpt1*, *Stat1*, *Agtr1a*, *Cdc42*, *Nox4*, and *Shc1* are differentially expressed in at least one time point (Figure 2-E). *Nox4* was reported to be upgraded in several kinds of DN models and played key factor in reactive oxygen species-related signal pathway [24–26].

To assess functions of differentially expressed genes, we performed gene ontology analysis for time-specific protein-coding genes (Figure 2-D, Materials). W4-specific gene functions are involved in phosphorylation, peptidyl-serine modification, and enzyme-linked receptor protein signaling pathway, whereas those for W8-specific genes are transmembrane transport, programmed cell death, oxidation reduction, and cell cycle.

To assess application of the mouse DN model, we validated differentially expressed genes by using human cell line *in vitro* diabetic model. We selected six differentially expressed genes with mono decreasing (increasing) gene expression and obtained their orthologous genes in human from Mouse Genome Informatics database. Figure 2-F shows expression levels of orthologous genes *in vitro* diabetic model by using qPCR. Surprisingly, all genes were differentially expressed after high-glucose stimulation and presented similar tendency (up- or downregulation) as that in mouse RNA-seq. These results indicated that mRNA data from RNA-seq in mouse DN model may provide similar evidence for human DN.

### Systematic profiling of lncRNAs during early development of DN

**Figure 3:**
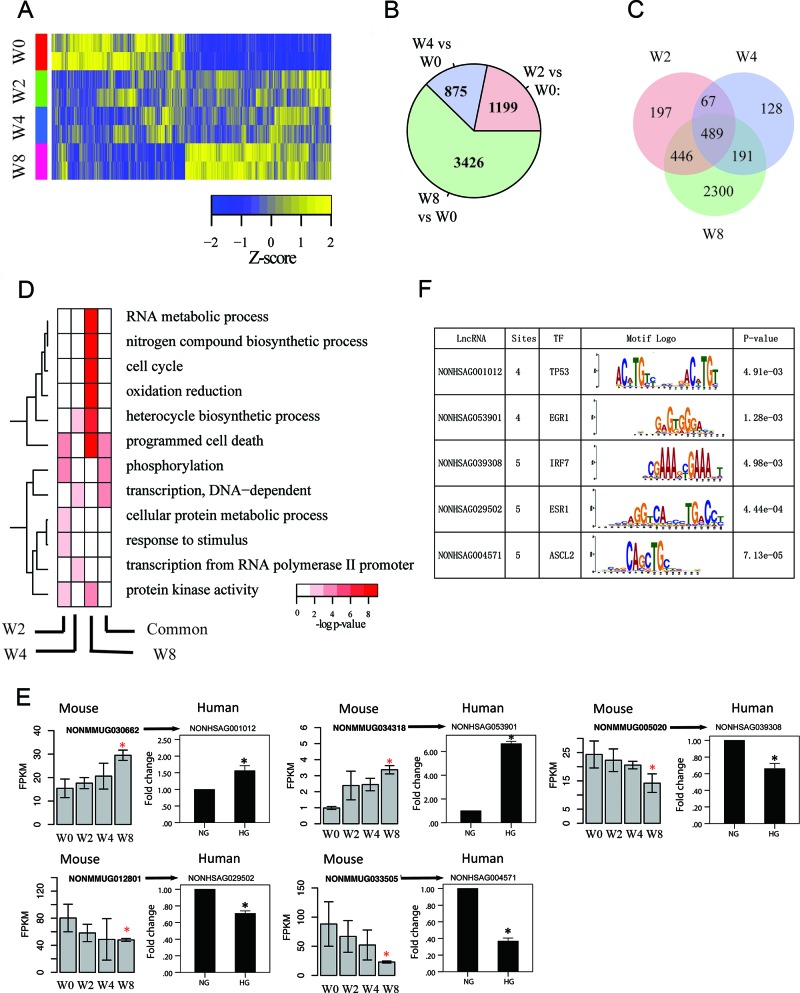
Transcriptome profiles of lncRNA genes associated with disease progression **(A)** Hierarchical clustering of lncRNA genes that are differentially expressed in at least one time point by comparison with that at W0; rows and columns correspond to samples and lncRNA genes, respectively. **(B)** Number of differentially expressed lncRNA genes at various time points compared with control (W0) with corrected *P*-value cutoff of 0.05. **(C)** Venn diagram of differentially expressed lncRNA genes at different times. **(D)** Gene ontology enrichment for week-specific and common differentially expressed lncRNA genes, presented as −log10 hypergeometric *P*-value for enrichment. **(E)** Validation for differentially expressed lncRNAs in PTCs (HK2)Left portion: Results from RNA-seq in mouse DN models (^*^p<0.05 versus W0). Right portion: Results from qPCR *in vitro* diabetic models. NG, cultured with normal glucose; HG, cultured with 30 mM glucose for 48 h. ^*^p<0.05 versus NG, *n*=3. **(F)** Motifs searched for validated lncRNAs in PTCs (HK2). TF, transcriptional factor. Sites, number of possible motifs in one LncRNA sequence.

We identified 29,273 lncRNA genes, of which 15,138 were expressed at FPKM value of ≥ 0.5 in at least one sample. Hierarchical clustering revealed that transcriptome profiles of kidneys were well separated during disease progression, and samples were clustered into three groups: W0, W2 and W4, and W8 (Figure 3-A). Figure 3-B indicated the largest number of differentially expressed lncRNA genes in W8 versus W0 comparison (*N*=3426, FDR<0.05) followed by W4 (W2) (*N*=875, 1199, FDR<0.05) (Figure 3-B). Figure 3-C shows overlapping of differentially expressed lncRNA genes, and the pattern is consistent with that for mRNA genes.

Neighbored protein-coding genes were used to analyze gene-ontology of time-specific differentially expressed lncRNA genes (Figure 3-D). Compared with protein- coding genes, lncRNA genes presented similar functions in gene ontology. However, comparison indicated that programmed cell death was at W2 for lncRNA genes, whereas function was enriched at W8 for protein-coding genes. Results suggested that dysfunction of pathways for kidney is initialized by disturbing regulators, such as lncRNA genes, beforehand.

To assess expression of orthologous lncRNA genes in humans, we used BLAST Software [27] to obtain orthologous lncRNA genes between mice and humans. We validated the expression of orthologous lncRNA genes in human cell line using qPCR (Figure 3-E). We validated the expression of orthologous lncRNA genes in human cell line. glucose stimulation and had similar expression pattern with orthologous genes of mouse. Considering these results, we concluded that lncRNA data from mouse diabetic model was also partly applicable to human DN.

Orthologous lncRNA genes in humans underwent motif searching (Figure 3-F). Motifs searching were calculated using TOMTOM software [28]. Surprisingly, some lncRNA has several possible motif sites for specific transcriptional factor binding. For instance, lncRNA NONHSAG053901 has four possible sites in its transcriptome for transcription factor (TF) early growth response protein 1 (EGR1)-binding. EGR1 was found to be responsible for activation of heparanase promoter under diabetic conditions [29]. Results implied that lncRNAs possibly directly interact with transcriptional factor and play important role in regulation of gene transcription or cell signal pathway.

### Differentially expressed lncRNAs are enriched by functional signals of ChIP-Seq

lncRNA genes are acknowledged as key regulators for diseases [30, 31]. To further characterize lncRNA signatures, we analyzed TSS of differentially expressed lncRNA genes for presence of histone modifications. Actively transcribed lncRNA genes were associated with promoter-associated histone modification (trimethylation of histone 3 at Lys4, H3K4me3) or enhancer-associated histone modification (monomethylation of histone 3 at Lys4, H3K4me1). Therefore, we used publicly available H3K4me1 and H3K4me3 ChIP-Seq data for kidney information from ENCODE database.

**Figure 4:**
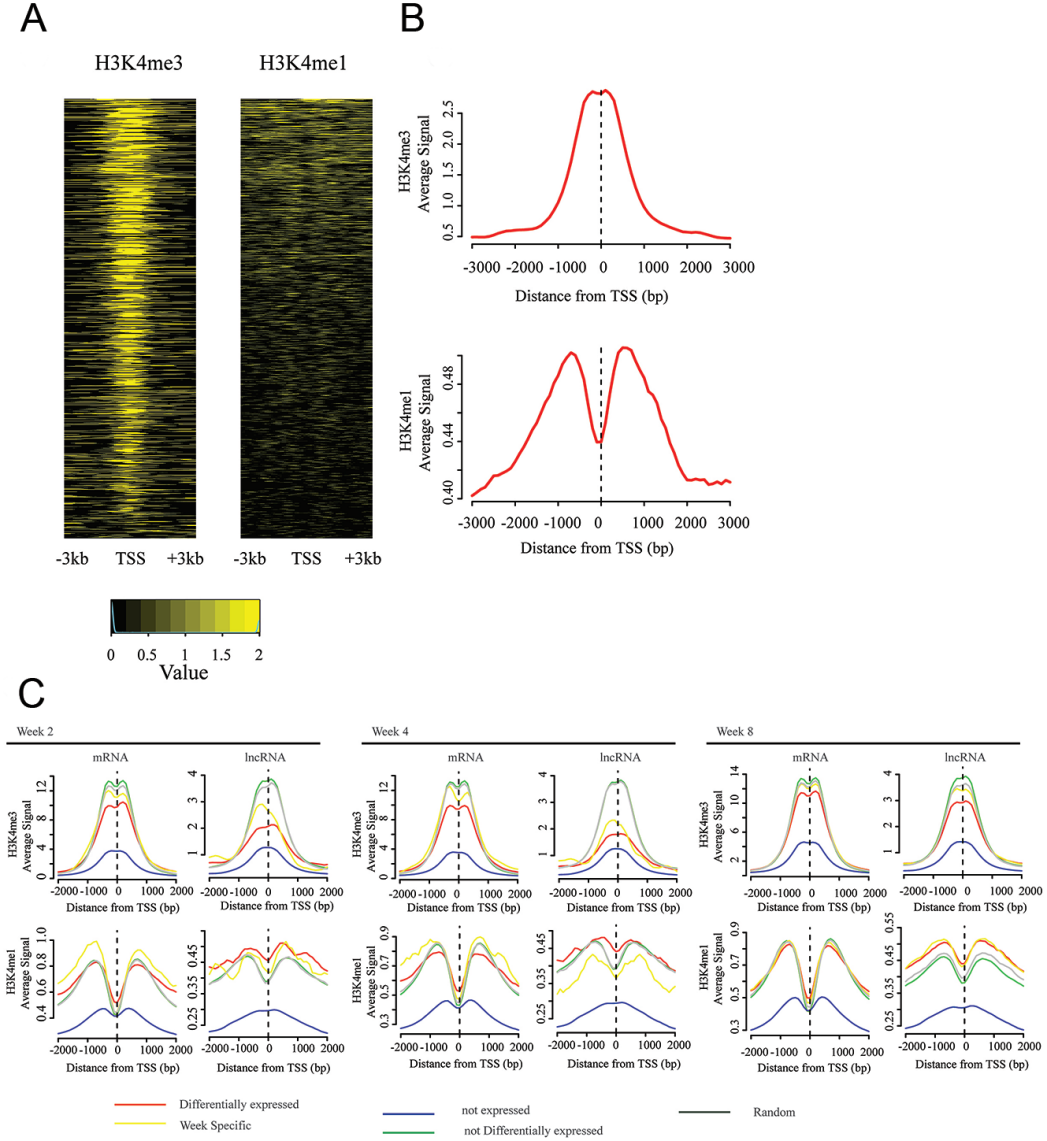
TSSs of differentially expressed lncRNA genes are enriched by active chromatin signal **(A)** ChiP-Seq analysis of histone-modification profiles (H3K4me1 and H3K4me3) at TSSs of lncRNA genes, which are differentially expressed in at least one time point. **(B)** Density functions for lncRNA genes of H3K4me1 and H3K4me3. **(C)** ChIP-Seq “metaplots” of histone-mark density at TSSs of protein-coding and lncRNA genes, which are stratified by gene expressions: differentially expressed genes, non-expressed genes, week-specific differentially expressed genes, expressed but not differentially expressed genes, and random genes.

Over one third of TSSs of differentially expressed lncRNA genes showed overlapping with significant peaks (39.7%, Methods and Materials) for at least one type of histone modifications, a proportion similar to that of previously annotated lncRNA genes associated with such histone modifications (37.5% [32], 37% [31]). H3K4me1 modifications showed distribution of broader density around TSSs than that of H3K4me3 modifications (Figure 4-A). Figure 4-B summarizes density functions of corresponding histone modifications. These results demonstrated that differentially expressed lncRNA genes enriched functional DNA elements.

We then determined difference among differentially expressed genes, non-differentially expressed genes, non-expressed genes, random transcripts for both protein-coding genes, and lncRNA genes for each time point; results are shown in Figure 4-C. In case of protein-coding genes, differentially expressed genes were associated with broader distributions of density of histone modification than non-differentially expressed genes at all time points. In case of lncRNA genes, similar tendency was observed at all time points. Comparison between protein-coding genes and lncRNAs indicated that differentially expressed lncRNAs had highest level of H3K4me3 modification among all lncRNA genes, where same observation was not observed in differentially expressed protein-coding genes (Figure 4-C).

In summary, our results indicate that transcriptional landscapes of mouse kidney were characterized by lncRNA genes.

### Expression signature of lncRNAs, but not mRNAs, characterizes DN progression

**Figure 5:**
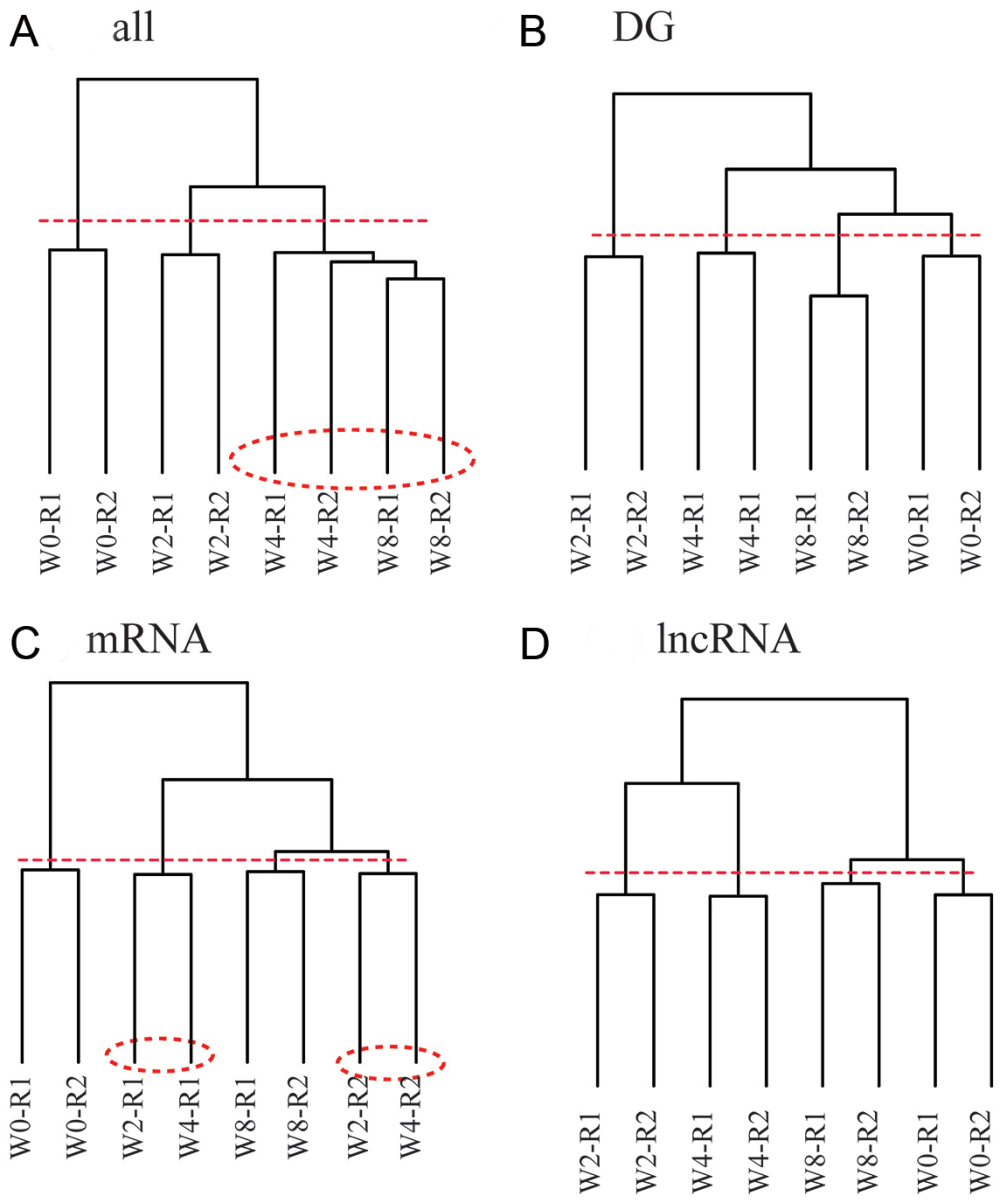
Disease progression is characterized by global expression patterns of lncRNA genes rather than mRNA genes Sample clustering analysis based on gene expression of various combination of protein-coding and lncRNA genes, where dashed line is cut line for dendrogram, and samples surrounded by dashed circles are misclassified. **(A)** Protein-coding and lncRNA genes. **(B)** Differentially expressed protein-coding and lncRNA genes in at least one time point. **(C)** Differentially expressed protein-coding genes. **(D)** Differentially expressed lncRNA genes.

Figure 3A suggests that lncRNA genes may serve as biomarkers in discriminating disease progression. Therefore, we clustered samples on the basis of both protein-coding genes and lncRNA genes (Figure 5-A). Branch containing samples of W0 and W2 were also well segregated, whereas W4 and W8 samples cannot be divided correctly.

We next constructed samples on the basis of differentially expressed protein-coding and lncRNA genes (Figure 5-B). Samples at all time points were correctly segregated when we cut the dendrogram at dashed line. Although samples were correctly classified, W2 and W4 samples were separated; this observation cannot be explained because disease progressed from W2 to W4.

To determine inaccuracy, we next constructed samples based on protein-coding genes that were differentially expressed in at least one time point (Figure 5-C). W0 and W8 samples were correctly classified, whereas W2 and W4 samples were mixed. Results indicated that protein-coding genes were inadequate for characterizing disease progression. We hypothesized that lncRNA genes can be used to characterize disease progression. To investigate inaccuracy factor, we next constructed samples based on the differentially expressed lncRNA genes (Figure 5-D). All samples were correctly classified when dendrogam was cut at the dashed line. W2 and W4 samples were also successfully segregated.

Overall, these data demonstrated that lncRNA expression can be used to define developmental relationships, while protein-coding genes are not.

### Correlation of lncRNA and protein-coding gene expression

**Figure 6:**
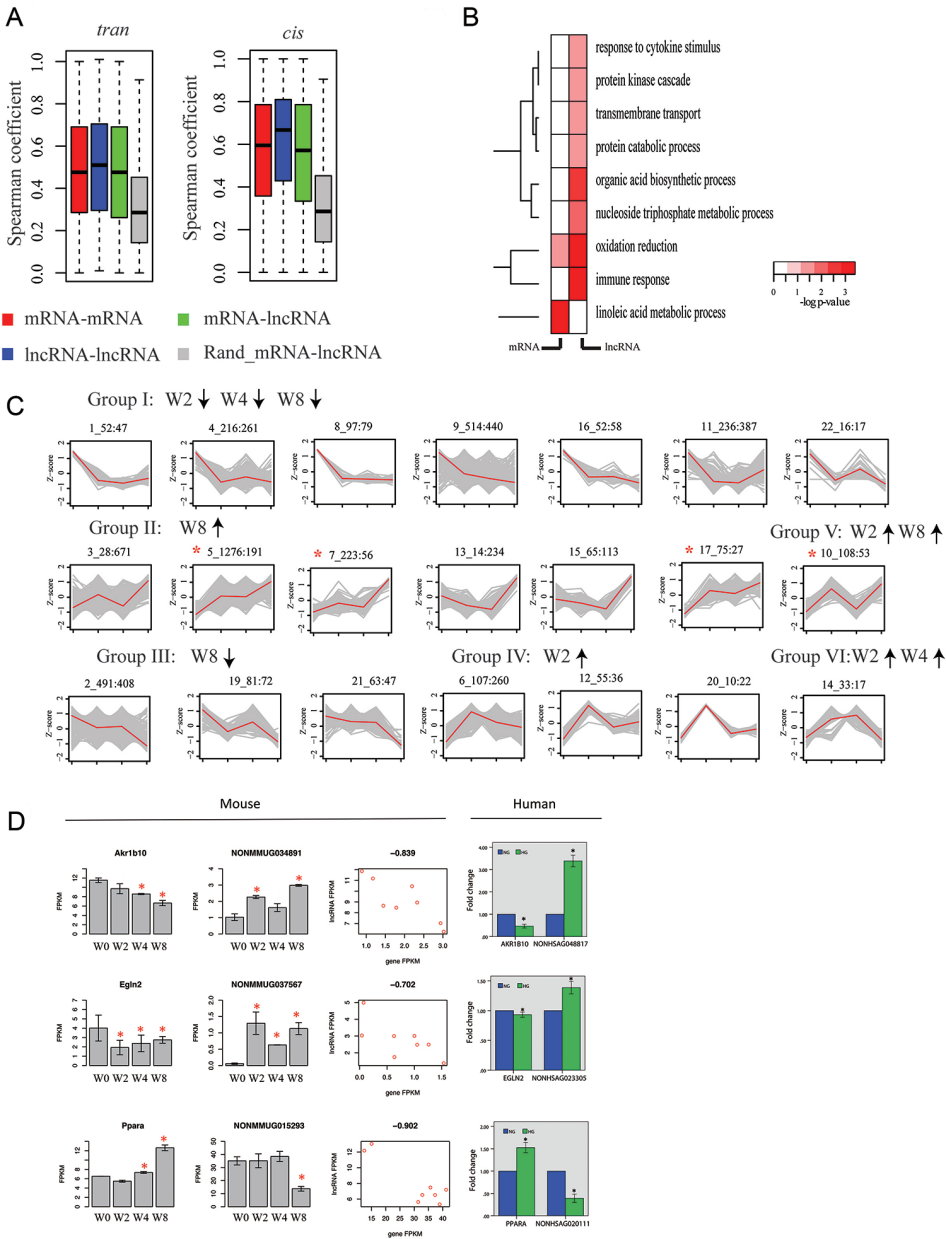
lncRNA genes are co-expressed with protein-coding genes during disease progression **(A)** Boxplot of pairwise Spearman expression correlations between genes of different classes in *trans* (left) and *cis* (right): lncRNA–to–lncRNA, lncRNA–mRNA, mRNA–mRNA, and random mRNA–lncRNA. **(B)** Gene ontology enrichment for protein-coding genes in *trans* and correlated with lncRNAs (first column, mRNA–lncRNA), and nearest protein-coding genes in *trans* and correlated with lncRNA genes (lncRNA–lncRNA) presented as −log10 hypergeometric *P* value for enrichment. **(C)** Model-based expression profiles (Profiles 1–22; Groups I–VI) of protein-coding and lncRNA genes expressed differentially during disease progression. Numbers above plots indicate profile number plus total genes and lncRNA genes in profile (in parentheses: total lncRNA). y-axis is Z-score of gene expression, and x-axis is time point. In each plot, gray line is expression level of gene in module, and red line is average expression level of genes within module. Red asterisks indicate profiles showing enrichment for lncRNA genes (*P* < 0.05 versus proportion of lncRNAs among all differentially expressed genes). **(D)** Validation for predicted mRNA–lncRNA pairs in PTCs (HK2) Mouse: Predicted mRNA–lncRNA pairs from RNA-seq in mouse DN models. Left portion: mRNA results (all differential expressions are significant). Middle portion: lncRNA results. Right portion: Correlation coefficient between mRNA and lncRNA. All *P* values < 0.01. Human: Validation for predicted mRNA–lncRNA pairs in PTCs (HK2) down-regulated lncRNAs by qPCR. NG, cultured with normal glucose; HG, cultured with 30 mM glucose for 48 h. ^*^p<0.05 versus NG, *n*=3.

To investigate co-expression patterns of lncRNA and protein-coding genes, we computed pairwise expression correlations across all RNA-Seq samples. We first analyzed *trans* correlations of expression (genes separated by distance of > 1M base or located on various chromosomes). Expression of lncRNA genes was more closely correlated than protein-coding genes in *trans*. In all cases, bias toward correlations was significantly higher than that obtained for control set of *trans* correlations, in which expression of lncRNA and protein-coding genes was randomly shuffled (Figure 6-A). We then analyzed cis correlations of expression (gene pair located within genomic window of 100K bases). We observed higher proportion of correlations among *cis* correlations than among *trans* correlation for both lncRNA-gene–protein-coding gene pairs and protein-coding gene–protein-coding gene pairs (Figure 6-A). These results indicate that lncRNA genes possibly perform functions via combination of multiple lncRNA genes.

Gene ontology enrichment analysis focused on protein-coding genes with strong *cis* correlations with protein-coding genes, and results revealed that enrichment for gene-encoding products involved oxidation and linoleic acid metabolic process. By contrast, genes with *cis* correlations with lncRNA genes were significantly associated with these functional annotations, such as response to cytokine stimulus, protein kinase cascade, and transmembrane transport (Figure 6-B).

To further investigate co-expression signature of genes, we delineated expression profiles of protein-coding genes and differentially expressed lncRNA genes in at least one time point (FDR<0.05). We used model-based gene clustering to obtain patterns for expression profiles [33], which are presented in Figure 6-C. In total, we discovered 21 modules, which were classified into six groups. Four specific profiles showed significant enrichment for lncRNA genes (*p*-value<0.05, proportion test) compared with proportion of lncRNA genes among all differentially expressed genes. These profiles were upregulated at W8 (profile 9, 10, 13, and 14), suggesting that more lncRNA genes became dysfunctional as disease progressed. Differentially expressed lncRNA genes showed significant enrichment (*P* < 0.05; relative to all lncRNA genes) for loci neighboring protein-coding genes, whose products were involved in transmembrane transport (*p*-value=9.7E-5, hypergeometric test), programmed cell death (*p*-value=8.4E-3), nitrogen compound biosynthetic process (*p*-value=5.6E-4), and cell cycle (*p*-value=1.0E-7). In summary, global co-expression analysis and gene-expression profiling suggested notable and previously unappreciated role for lncRNAs in early development of DN.

To assess correlation of lncRNA and protein-coding genes, we validated gene expression of orthologous genes in humans using qPCR (Figure 6-D). We showed that three typical pairs of lncRNAs and protein-coding genes were consistent with those trends in mice.

### Co-expression network analysis reveals modules of lncRNA and protein-coding genes

Correlation-based approaches are widely used to infer function of lncRNAs. Thus, we used network analysis by using WGCNA algorithm [34], which identified modules of genes with strong co-expression. Eigengene measures expression profile of co-expression module, and such profiles can be used to rank individual genes within modules. Screening for genes with high module membership is useful strategy for identification of genes of interest.

**Figure 7:**
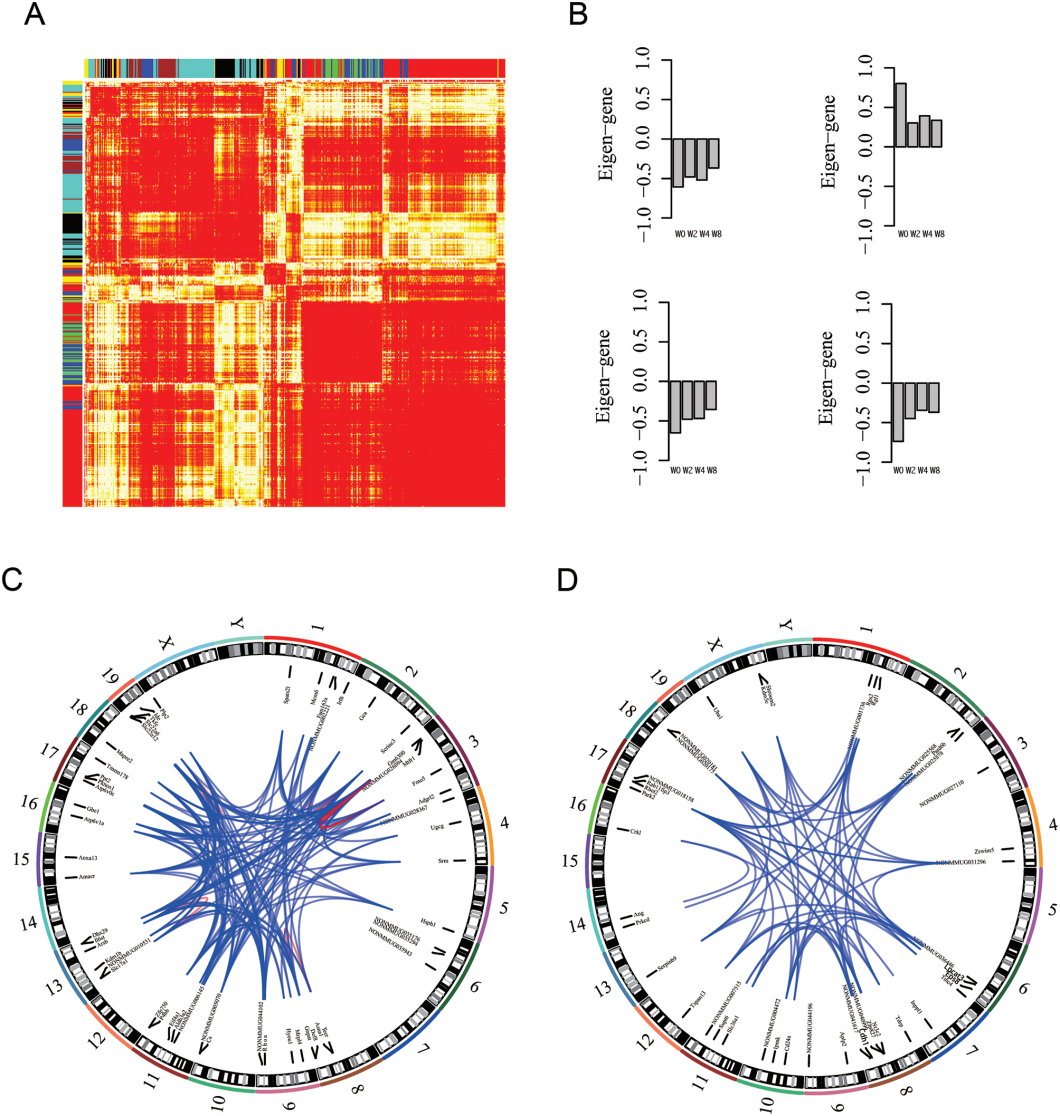
Network analysis identifies co-expression modules of lncRNA and protein-coding genes **(A)** Heatmap of WGCNA topological overlap matrix, where each row (column) corresponds to a gene, and bar color indicates genes clustered as module. **(B)** Eigengene of selected modules represents gene expression level of genes within a module. **(C-D)** Screening strategies for identification of candidate lncRNA genes and lncRNA-gene–protein-coding gene co-expression associations in specific modules for further functional studies presented as Circos plots from analysis of HSPC module (broken lines in circumference indicate individual chromosomes).

Of 102 modules discovered by WGCNA, four modules were selected for further analysis. Figure 7-A shows heatmap of protein-coding and lncRNA co-expression network. As shown in Figure 7-B, in eigengenes of four selected co-expressed modules, expression level of modules mono-decreased (increasing), indicating disease progression. These modules contained protein-coding genes associated with cell functions, including transmembrane transport (*p*-value=0.01), lipid biosynthetic process (*p*-value=7.2E-4), oxidation reduction (*p*-value=0.01), and cell division (*p*-value=8.7E-3). Figure 7-C provides two schematic examples of co-expression module consisting of protein-coding and lncRNA genes, and functions of lncRNAs can be inferred from protein-coding genes within modules based on topology. Overall, these results provide information for identification of lncRNA candidates and formulation of hypotheses for functional studies to elucidate role of lncRNAs in DN.

## DISCUSSION

Discovering new biomarkers and therapy targets is crucial for diagnosis and treatment of DN. Progress, however, is slow. Recently, research on lncRNAs illuminated understanding of DN. Few lncRNAs have close relationship with DN progression; these lncRNAs include lncRNA PVT1 [18], lnc-MGC [35], and Tug1 [19], etc.. Difficulty arises from exposing complete structure of lncRNAs in DN process these dispersive studies, and our understanding is presently in its infancy. Thus, large-scale data on lncRNAs and mRNAs in DN need to be gathered for better understanding of this disease. Recently, Ding et al [36] investigate lncRNA expression in DN using the microarray data. However, they use the mixed cells from kidney, rather than the PTCs. Hence, we revealed genome-wide profiles of lncRNAs and mRNAs from isolated fresh PTCs using NGS in DN process for the first time. Transcriptome information from fresh isolated tissues can represent actual appearance of disease to maximum extent [37]. Therefore, this study is novel and pioneering on global views about lncRNAs and mRNAs in DN research.

To our knowledge, this report represents first investigation of protein-coding and lncRNA elements in mouse kidney PTCs during early development of DN. And, we discovered more than 3,502 differentially expressed protein-coding genes, in which 76 genes are reported in previous studies. We also identified 3,818 differentially expressed lncRNA genes. Interestingly, 390 (556) lncRNA genes exhibited mono-decreasing (mono-increasing) expression levels. Patterns indicate dynamics of genes during disease progression, suggesting important role of lncRNAs during initial stages of diabetes. Functions of lncRNA genes include cell proliferation and cell death, and they are closely associated with diabetes.

Prior information was used to annotate lncRNA genes. Recently, Orom et al. [38] used GENCODE annotation of human genome to characterize over a thousand lncRNAs, and discovered that significant portion of lncRNAs have *cis*-regulatory enhancer properties. To characterize the differentially expressed lncRNA genes, we analyzed TSSs of lncRNAs for presence of histone modifications. By using ChiP-Seq data (H3K4me1, H3K4me3) from ENCODE database for kidney [39]. And, we discovered that nearly 40% of differentially expressed lncRNA genes overlap with histone modification signals. Result demonstrates that obtained lncRNA genes are key regulators for kidney disease. Moreover, we showed that lncRNA genes are more accurate than mRNA genes in discriminating disease progression. These results are consistent with previous study [31], where lncRNAs discriminate cell development. Functions of lncRNA genes are cell proliferation, cell death, oxidation reduction, and phosphorylation, and these are closely associated with DN. In addition, current study indicates that lncRNA genes exhibited earlier symptoms of disease compared with that of mRNA genes, which may provide novel evidence for early diagnosis and therapy of DN.

To further investigate application values of mouse model, we validated several that human orthologous lncRNAs *in vitro* diabetes model. And, they had similar differential expression pattern as their orthologous lncRNA in mouse. Interestingly, the lncRNAs have not been reported in DN before. Poor conservation of lncRNAs across different species challenges application of animal sequencing data to human disease [6, 40]. However, in clinical practice, in patients with early DN, conditions prevent obtaining sufficient kidney samples for deep sequencing. Our study demonstrates that validation of orthologous lncRNAs from animal data is still an efficacious way to discover novel biomarkers in human DN.

lncRNAs exert their effect by direct interaction with specific TFs [41, 42]. In recent report, hypoxia-regulated lncRNA linc-p21 was shown to physically interact with HIF-1-alpha TFs to control metabolism in tumor cells [43]. We discovered several enriched important TF motifs for lncRNAs. These TFs were previously reported as key regulation factors that closely related to cell proliferation, apoptosis, inflammation, fibrosis, and other signaling pathways in DN [29, 44–46]. In our study, some of predicted potential lncRNAs–mRNA pairs have also been validated in in human cells *in vitro* diabetic model. Results imply that orthologous lncRNAs may play similar roles in DN even across different species.

## MATERIALS AND METHODS

### Animals

Eight weeks old male DBA/2J mice were purchased from Jackson Laboratory (Bar Harbor, ME) and were housed in barrier facility under National Institutes of Health guidelines. The mice were divided randomly into four groups of 48: 0 week group(Control, twelve mice); 2 week group(Diabetes, twelve mice); 4 week group(Diabetes, twelve mice) and 8 week group(Diabetes, twelve mice). In each group, mixed PTCs from every six mice were used for producing one RNA-sequencing dataset. Thus, each group produced two independent RNA-sequencing datasets. Diabetes was induced with intraperitoneal injections of streptomycin (STZ) based on protocol of Animal Models of Diabetic Complications Consortium [47]. In brief, STZ (Sigma) was dissolved in 0.05 M citrate monosodium (ACROS) buffer immediately before injection. Mice received injections of 50 mg/kg/day STZ for five consecutive days after fasting for 4 h. Low doses of STZ has been previously demonstrated to induce durable hyperglycemia without obvious kidney damage [48, 49]. All mice were fed the same diet and water. Insulin was not given to any of the animals. All animal procedures were approved by the Institutional Animal Care and Use Committee of Sichuan University.

### Blood glucose and urinary albumin/creatine ratio measurements

Mice blood glucose levels were measured per week after initial STZ injection using ReliOn Ultima glucose reader (Walmart) for 10 weeks. At each time point before sacrifices, spot urine collections were obtained from mice as previously described [50]. Urinary concentrations of albumin and creatine were determined using mouse-specific microalbuminuria enzyme-linked immunosorbent assay kit (Albuwell M, Exocell) and creatinine companion kit (Exocell), respectively, per manufacturer's instructions [51].

### Histology

Kidney tissues of mice were first placed in 4% paraformaldehyde and then embedded in paraffin. Paraffin-embedded tissues were then sectioned at 3 μm to 5 μm, stained with hematoxylin-eosin, periodic acid–Schiff (PAS), and Masson's trichrome, successively. Digital images were obtained using Leica DM5000B Microscope System. For each mouse, 10 randomly selected cortical areas were evaluated under 200× magnification. Relative interstitial volume was evaluated by morphometric analysis, and tubules were evaluated as tubules damage index (TDI) to estimate percentage of damaged tubules in blinded manner as previously described [23].

### Fluorescence-activated cell sorting (FACS)

The cortical region of mice kidneys was minced into small pieces. Cortical pieces were softly grinded on 250 μm steel sieve, washed with 1×PBS, and spun down. Precipitation was resuspended in solution of 0.5 mg/ml collagenase I, 0.5 mg/ml dispase II, and 0.1% trypsin (Worthington Bio Co., Lakewood, NJ) in complete RPMI-1640 medium (Gibco) and incubated at 37°C for 15 min. After digestion, mixture was filtered through 100 and 40 μm cell strainer (BD Biosciences) sequentially. Single cell suspension was treated with ACK lysing buffer (Lonza), then resuspended in 1×PBS to 2×10^7^ cells/ml. Suspension was first incubated with anti-CD16/32 antibody (3 μl/1× 10^8^ cells) (Biolegend) at 4°C for 10 min. Then cells were washed and resuspended. Kidney cells were incubated with PE-conjugated CD13 antibody (BD Biosciences), which is specific against aminopeptidase N on PTC microvilli (20 μg/ml) at 4°C in dark for 30 min. A647 conjugated nephrin antibody (Bioss) was added to prevent podocyte contamination. After labeling, cells were washed and resuspended in 1×PBS supplemented with 2% bovine serum albumin to 1×10^7^ cells/ml. Fluorescent-labeled PTCs were isolated by BD FACSAria™ II flow cytometry cell sorting system (BD Biosciences). [52].

### RNA-seq

Sorted PTCs were lysed with Trizol-LS immediately (Ambion), and total RNA was prepared using RNeasy Plus Mini Kit (Qiagen) per manufacturers’ instructions. Total RNA concentration was measured by Qubit 2.0 Fluorometer (Invitrogen), and RNA quality was evaluated using Agilent 2100 Bioanalyzer (Agilent). RNA-seq libraries were prepared with Ovation RNA-Seq System V2 Kit (NuGEN) per manufacturer's instruction. RNA-seq libraries were analyzed by Bioanalyzer (Agilent) and quantified by quantitative PCR (qPCR) (KAPA). High-throughput sequencing was performed using Illumina HiSeq 2500 Genome Sequencers [53]. The data is available at GEO database with accession number GSE95367.

### Cell culture

Human kidney epithelial cells (HK2) of proximal tubular origin were purchased from American Type Culture Collection (Manassas) and cultured in RPMI 1640 medium (Gibco) supplemented with 10% fetal bovine serum (Gibco) at 37°C under 5% CO_2_. Cells were cultured in complete medium with different concentrations of glucose for further validation experiments at 80% confluence after synchronization.

### Real-time qPCR for mRNA and lncRNA expression

Total RNA was purified from HK-2 cells using RNeasy Plus Mini Kit (Qiagen) as described ahead. cDNA was obtained by reverse transcription using iScript cDNA Synthesis Kit (Bio-Rad). Then, cDNA was amplified with specific primers (shown in Supplementary Tables 1&2) and detected using SYBR Green Supermix (Bio-Rad) with BIO-RAD CFX-96 Real Time PCR System (Bio-Rad) under the following conditions: 95°C for 3 min followed by 40 cycles of 95°C for 10 s and 52°C for 30 s. Amount of relative expression of cDNA was calculated using 2^−△△Ct^ method. Expression levels were normalized to glyceralde-hyde-3-phosphate dehydrogenase mRNA as internal control.

### Data analysis

#### Transcriptome assembly and expression level estimate from sequencing reads

Paired-end reads were mapped to mouse genome (mm9) using Tophat [54]. Downstream analyses only included uniquely mapped reads with fewer than two mismatches. Transcripts were assembled using Cufflinks [55] and Refseq (mm9) as source of annotated transcripts. Normalized transcript abundance was calculated using Cufflinks. Fragments Per Kilobase of transcripts per Million mapped reads (FPKM) was used to quantify expression (The procedure for the RNA-sequencing analysis is depicted in Supplementary Figure 3). Gene-level FPKM values were computed by summing up FPKM values of their corresponding transcripts. For lncRNA gene expression, same procedure was repeated by using annotation file of NONCODE 4.0 [56] (FPKM values for mRNA are presented in Supplementary 3, and FPKM values for lncRNA are shown in Supplementary 4).

#### Statistical significance of differential gene expression

With normalized gene level read counts, edgeR [57] was adopted to calculate *p*-values of differential gene expression. For calling differentially expressed gene, we used false discovery rate (FDR) cutoff of 0.05, and minimum FPKM is greater or equal to 1 in at least one sample.

#### Gene ontology enrichment analysis

To evaluate functional relevance of genes, we performed gene ontology enrichment analysis on groups of genes using hyper-geometric test. *p*-values were corrected through BH test [58] with cutoff value of 0.05.

#### Chip-sequence data analysis

We downloaded Chip-Seq datasets (Encyclopedia of DNA Elements at UCSC [39]) of H3K4me1 and H3K4me3 histone modifications in kidney from eight-week old adult mouse. We used peak information from bigWig files. For various genes, significant peaks and transcription start sites (TSS) overlapped under conditions of strict overlap (TSS was contained in peak region as defined by MACS [59]). Data for read density heatmaps were obtained for window sizes ±3 kb.

#### Expression correlation analysis

We calculated correlations for mRNA–mRNA, mRNA–lncRNA, and lncRNA–lncRNA pairs. For each pair of genes with non-null expression, non-parametric Spearman correlation was computed using FPKM expression. Both *trans* (pairs consisting of genes located at distance of > 1 Mb from each other or in different chromosomes) and *cis* (pairs consisting of genes located within genomic window of 1Mb) correlations were computed. Controls used were correlations of lncRNAs and mRNAs with randomly shuffled expression vectors.

#### Co-expression network analysis

The protein-coding and lncRNA genes differentially expressed at least on one condition were selected for co-expression network analysis (protein-coding: 3884; lncRNA: 3997). Weighted gene co-expression network analysis algorithm (WGCNA) was adopted with default parameters [34].

Pair-wise co-expression matrix (Topological Overlap Matrix) was generated and clustered to retrieve modules of highly correlated genes (The dendrogram is illustrated in Supplementary Figure 4). Each module obtained by WGCNA was characterized by expression of representative gene (eigengene, Figure 7-B). For each gene, WGCNA also provides summary statistics and module membership (quantifying how strongly expression profile of gene correlates with that of module) information, which can be used to screen for biologically interesting candidate genes for functional studies.

## CONCLUSIONS

Overall, novel findings in this study unveiled largely unknown field of lncRNAs in early stage of DN. At least four light spots were revealed in current study. First, we used NGS for transcriptome deep sequencing of fresh isolated kidney cells from dynamic process in early DN for the first time. Thus, research methods are advanced and groundbreaking. Second, algorithms of big data are adequately scientific and reliable. Third, data acquired from mouse were proven to be applicable by validation in human cells. Fourth, deep bioinformatics analysis for validated lncRNAs is the pilot for future concrete mechanism research. In conclusion, present resource paves new way for aiming at lncRNAs in early diagnostic and intervention strategies of DN.

## SUPPLEMENTARY MATERIALS FIGURES AND TABLES









## Abbreviations

LncRNA: long non-coding RNA
DN: diabetic nephropathy
H3K4me1: histone H3 lysine 4 monomethylation
H3K4me3: histone H3 lysine 4 tri-methylation
WGCNA: weighted gene co-expression network analysis algorithm
TSS: transcription start sites
FPKM: fragments Per Kilobase of transcripts per Million mapped reads
FACS: fluorescence-activated cell sorting.
