# Supplementary material for: RNA-sequencing reveals genome-wide long non-coding RNAs profiling associated with early development of diabetic nephropathy
Source: Oncotarget. 2017 Nov 11;8(62):105832–47. doi: 10.18632/oncotarget.22405 (PMC5739683; doi:10.18632/oncotarget.22405)
Supplement: Supplementary file 1 [file oncotarget-08-105832-s001.pdf]

RNA-sequencing reveals genome-wide long non-coding RNAs profiling associated with early development of diabetic nephropathy

SUPPLEMENTARY MATERIALS

Post-FACS purity analysis

Purity of sorted cells was re-analyzed by flow cytometry using PE labeled aminopeptidase antibody. Over 99.0% positivity ensures the accuracy of the experiment. The PE positive population was shown on the right panel.

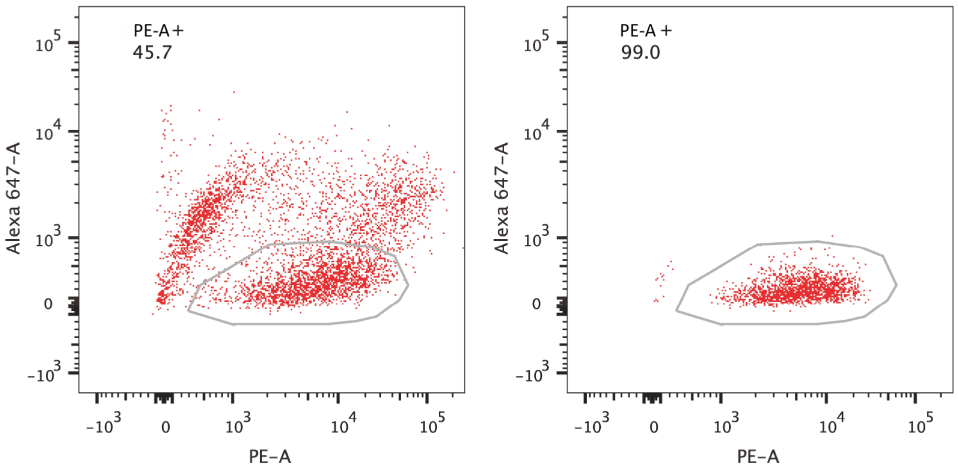

Supplementary Figure 1: Post-FACS purity analysis. The cell group circled with gray line in the right figure is PE positive.

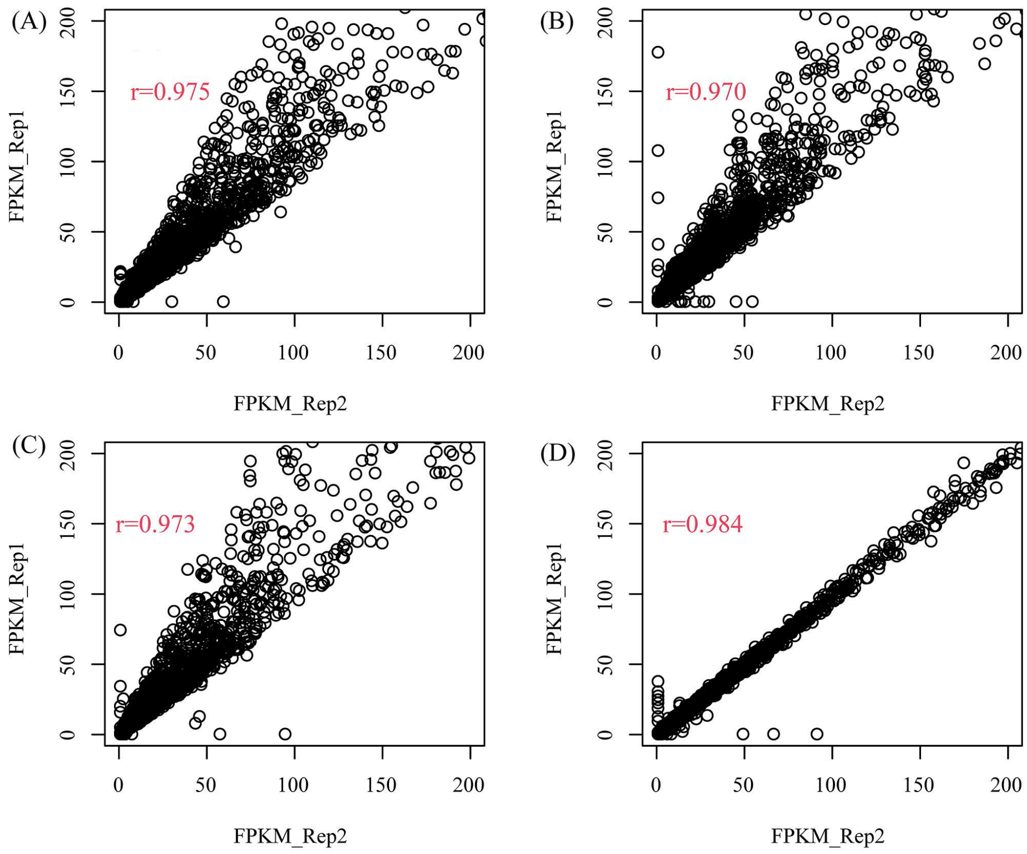

Supplementary Figure 2: Inter-replicate concordance.

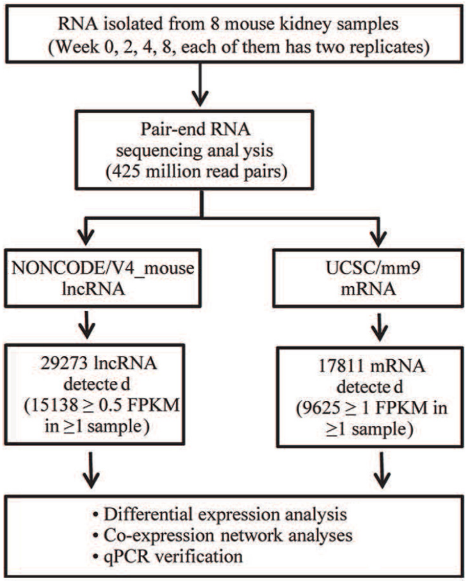

Supplementary Figure 3: Pipeline for analyzing the RNA-seq data.

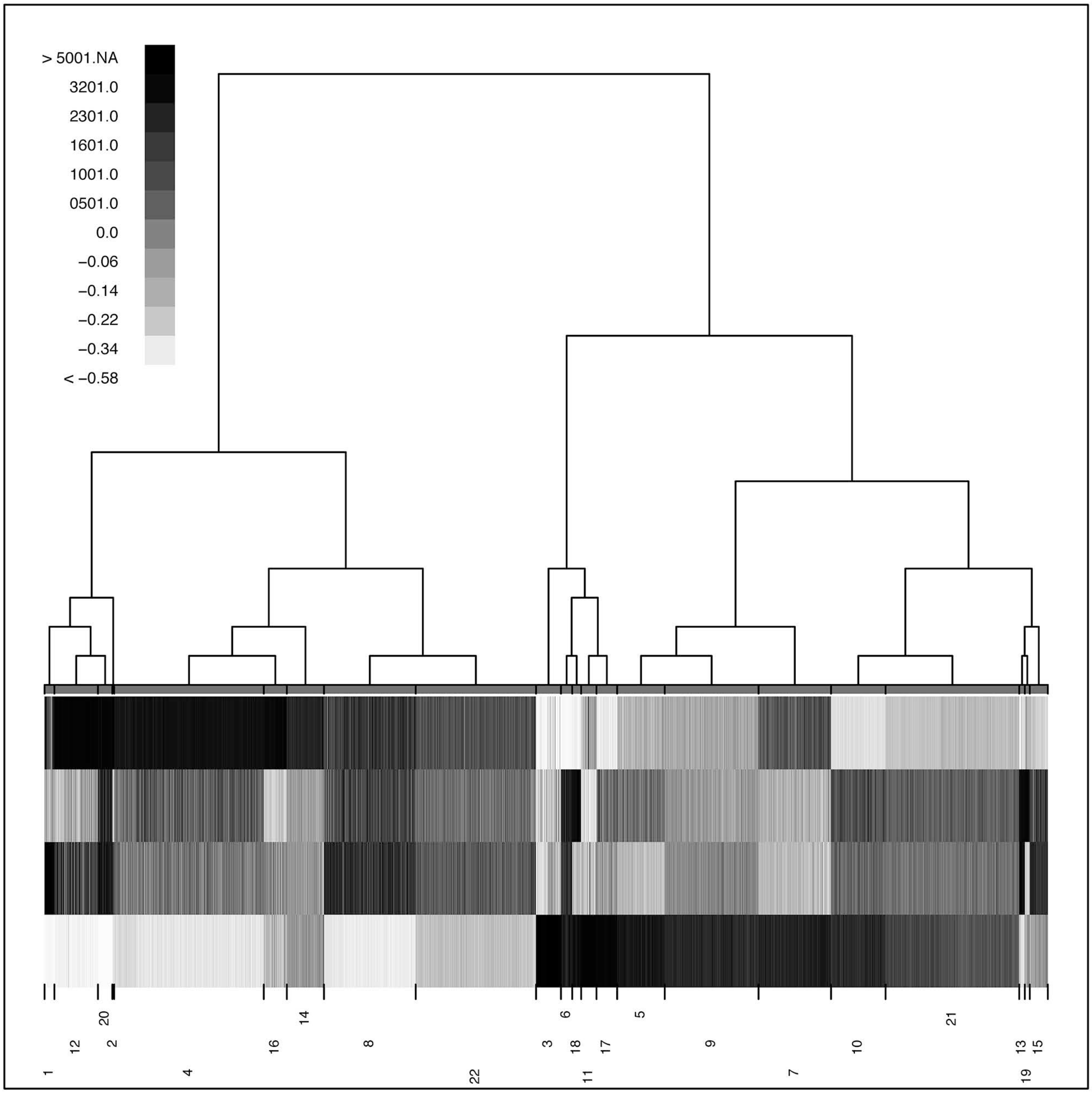

Supplementary Figure 4: Dendrogram of protein-coding and lncRNA correlation modules.

Supplementary Table 1: mRNAs Primers for validation in human cells

mRNA	Forward	Reverse
**Akr1b10**	ACTTCTACCTGCTCACTCA	TGCTTCTTTCACTTTGC
**Egln2**	GAATCAGAACTGGGACG	CCAGAAAATGAGCAACC
**Gclm**	TGGCTTTTAGTCCTTCCT	ATGTTGGGATACTGTGGG
**hps3**	CCCCTGGAACTTCTTGGT	GGTGCTGAAAGTGGGAAT
**Ifi27**	CCTCCATAGCAGCCAAGA	ATGGAGCCCAGGATGAA
**npm1**	CTACCTAAGTGCGTGCCG	CTGGTGCTCATTTTCATCAT
**Ppara**	CGGCGAGGATAGTTCT	GGGGACCACAGGATAAG
**Ppia**	AACCCCACCGTGTTCTTC	GCTACTACCTCCAATCTGTTA
**GAPDH**	CTGCCACCCAGAAGACT	CAGTGAGCTTCCCGTTC

Supplementary Table 2: LncRNAs Primers for validation in human cells

LncRNAs	Forward	Reverse
ONHSAG001012	AATGGACATTAGGACCCG	AGAATGCCCACGGAATAA
NONHSAG004571	TCTGCTAAACCTGCTCCT	TTTGGCATCTCCATTTTC
NONHSAG020111	CTGGCTGCGACATCTG	CTCTATCGGGGTTGGT
NONHSAG023305	TGAAGGCATTGTTATCC	CATCATCAGAACCCAAA
NONHSAG029502	CAAGTGACATCCTCCAAAAT	TCTTCTAACTGCTTGCCTAC
NONHSAG039308	CTTACATTCAGTTGTCTTGGAG	CCGTTCTTTTATGCTTGG
NONHSAG048817	TCCCTCTGCGAGTTCA	CCTGCAAGCATCATCAAC
NONHSAG053901	GGAAGGAAGGAGGTGGG	TGCGGTACGAGGAAACA
GAPDH	AGTCCACTGGCGTCTTCA	GAGGCTGTTGTCATACTTCT

Supplementary Table 3: The reported genes associated with diabetic nephropathy

See Supplementary File 1

Supplementary-3: The FPKM values for the mRNA genes.

See Supplementary File 2

Supplementary-4: The FPKM values for the long noncoding RNA genes.

See Supplementary File 3
